# Occurrence of Injuries in Different Phases of Judo Matches: Analysis Based on International Competitions

**DOI:** 10.3390/sports12120354

**Published:** 2024-12-19

**Authors:** Wiesław Błach, Dawid Gaweł, Wojciech J. Cynarski, Łukasz Rydzik, Zbigniew Borysiuk, Maciej Kostrzewa

**Affiliations:** 1 Faculty of Physical Education & Sport, University of Health & Sport Sciences in Wroclaw, 50-375 Wrocław, Poland; wieslaw.judo@wp.pl; 2 Institute of Sport Sciences, Jerzy Kukuczka Academy of Physical Education, Mikołowska 72a, 40-065 Katowice, Poland; d.gawel@awf.katowice.pl; 3 Institute of Physical Culture Studies, College of Medical Sciences, University of Rzeszow, 35-310 Rzeszow, Poland; ela_cyn@wp.pl; 4 Institute of Sports Sciences, University of Physical Education, 31-571 Kraków, Poland; 5 Faculty of Physical Education & Physioteraphy, Opole University of Technology, 45-758 Opole, Poland; z.borysiuk@po.edu.pl

**Keywords:** combat phases, injury analysis, judo competition, grappling, injury prevention

## Abstract

Background: The specificity of the technical actions that are performed in judo may be dependent on and vary during particular phases of the combat, thus possibly impacting injury prevalence. Therefore, towards the betterment of athletes’ safety and considering the importance of the issue of injury prevention in judo, the main goal of this study was to identify the injury occurrence during respective combat phases, i.e., the (a) first half, (b) second half, (c) last minute, and (d) golden score. Methods: A total of 26,862 elite judo athletes, including 15,571 men and 11,291 women, participated in the study. The subjects competed in 128 international tournaments of the European Judo Union (EJU) in 2005–2020. The EJU medical questionnaire was utilized to gather information from each injured athlete according to the aforementioned inclusion criteria. Results: Several statistically significant relationships were found between the differences in the number of injuries between particular combat phases and the athlete’s sex. Conclusions: A significantly higher number of injuries occurred during the combined second half and the last minute of the judo combat. Males suffered more injuries during the first and second half of the combat compared to females, however, during the last minute of the combat, the number of injuries was greater among women.

## 1. Introduction

Judo is one of the most prominent martial arts in the world, as well as an Olympic combat sport, alongside wrestling, boxing, taekwondo, and karate [1,2,3,4]. It is characterized by high-intensity intermittent actions, and requires excellence in technical and tactical preparation, as well as well-developed multiple motor abilities, including strength, power, and endurance [5]. In recent years, in both the male and female categories, the number of its participants has grown exponentially. The increasing number of practitioners with concomitant increments in injury occurrence raises a significant issue regarding the health and safety of these athletes. Judo has been shown to be a high-injury-risk activity, with significantly higher injury rates per 1000 h of activity (16.3) compared to other martial arts such as wrestling (9.1) and karate (6.7) or even popular team sports such as soccer (7.8) and volleyball (7.0) [6]. The most severe injuries, regardless of performance level in judo, with respect to time loss and reduction in performance, are anterior cruciate ligament ruptures and vertebral disc prolapses [7]. It is interesting to note that, even among Olympic combat sports, judo, in parallel to boxing and taekwondo, presents the highest injury rate [4]. Furthermore, it must be noted that the injury incidence and characteristics may differ between judo practice and competition, therefore, the data related to judo practice cannot be extrapolated to competitions [8]. Although injury prevention is unattainable in competitive sport, all the available measures must be taken in order to decrease the possibility of injury occurrence. Therefore, the International Judo Federation implemented new regulations aiming towards the betterment of athlete safety, which have changed several times over the last two decades [8,9,10]. Moreover, besides changes in the official regulations, researchers and practitioners have also raised the need to identify the risk factors which may lead to refined injury prevention tactics and performance [11], as well as improved preparation of medical personnel and the organizers of the tournaments, which, in turn, may lower the burden of exclusion from training and competition among athletes [4,8].

The specificity of the technical actions that are performed in judo may impact the characteristics and nature of injury prevalence in judo. During judo combat, an athlete strives to move the center of gravity of the opponent out of the axis [3,12]. Thus, when the weight of the opponent is greater, the difficulty of this task increases. Therefore, the weight of the opponent should be considered as one of the factors impacting injury risk in judo. It has also been shown that the highest number of injuries and attendance frequency before and after fights occurred in the absolute (open weight and no age limit) category when compared to the adult and juvenile categories [13]. Further, competition in weight-equated categories has been reported to decrease the occurrence of injuries [3]. In adult categories compared to juvenile ones, higher percentages of injuries arise, which may be explained by the smaller number of athletes, in general, in this category compared to juvenile categories. Besides age and weight categories, judo injuries may be considered in regard to the type of action, i.e., standing fight (tachi-waza) or ground fight (ne-waza). It has been shown that more injuries occur during standing fights (tachi-waza) compared to ground fights (ne-waza [13,14,15]. Furthermore, most injuries occur during falling and throws, thus, defensive (uke) athletes are more likely to suffer an injury compared to attacking (tori) athletes, due to athletes’ need to protect themselves from falling on their back during defensive maneuvers [16]. It should also be noted that some studies have suggested that sex may also influence the rate of injuries among judo athletes, due to differences in the fighting style and motor ability levels between the sexes, as well as hormonal fluctuations. However, the data in this regard still remain contradictory [15].

Despite the growing body of research in regard to injury epidemiology during judo contests, a recent systematic review by Mooren et al. [8] highlighted the heterogeneity and inconsistency of current reports. Furthermore, still there is no uniform definition of injury, as some studies do not take injury severity into account (regardless of the need for medical attention or time loss), while some do [8,17]. Beyond the aforementioned factors influencing the risk of injury in judo and the plethora of studies related to the time structure of judo combat in regard to physiological and perceived responses, as well as time motion measures, to the best of the authors’ knowledge, the literature has not yet examined the impact of the time structure of a singular judo contest in regard to injury prevalence. Given this, standing (tachi-waza) and ground (ne-waza) fights result in different energy demands [18], as well as specific actions in judo (i.e., attack/defense, grip attempts, groundwork combat may be associated with specific combat phases (due to increasing fatigue levels)), so such investigation may be warranted [8,19].

Therefore, towards the betterment of athlete safety and considering the importance of the issue of injury prevention in judo, the main goal of this study was to analyze and identify the injury occurrence during respective combat phases, i.e., the (a) first half, (b) second half, (c) last minute, and (d) golden score, among males and females. Taking into account the fact that specific actions in judo may be associated with specific combat phases, as well as physiological modulations in metabolic and inflammatory parameters alongside developing fatigue [20,21,22], we hypothesized that the majority of injuries would occur during the second half of the combat. It was also hypothesized that, due to differences in the fighting style (ratio of standing (tachi-waza) and ground (ne-waza) fights) and tactics between sexes [18,19], the occurrence of injuries during particular combat phases would differ between sexes.

## 2. Materials and Methods

### 2.1. Study Participants

A total of 26,862 elite judo athletes, including 15,571 men and 11,291 women, participated in the study. A power analysis was conducted prior to data collection to determine the required sample size for detecting statistically significant differences in injury occurrence across the combat phases, accounting for gender and injury severity. Based on an expected moderate effect size (Cohen’s *d* = 0.5, *d* = 0.5), a significance level of *p* < 0.05, *p* < 0.05, and a desired power of 0.80, the minimum sample size required was calculated to be approximately athletes. The actual sample size of 26,862 athletes far exceeded this requirement, ensuring that the study was adequately powered to detect meaningful differences in injury patterns and timing. The inclusion criteria were as follows: elite able-bodied judo athletes (both male and female), aged between 19 and 35 years, with a minimum of 3 years of elite-level judo training experience, and participating in at least two judo training sessions per week. Injuries were included if they were officially recorded in the European Judo Union (EJU) medical database during the competition period and diagnosed by a licensed sports physician present at the event. Injuries were classified as either common (e.g., nosebleeds, bruises, and abrasions) or serious (requiring immediate medical intervention and resulting in fight discontinuation). The exclusion criteria included judo athletes (male or female) below the age of 19, athletes with physical disabilities, injuries reported without verification by a sports physician or lacking proper documentation, chronic injuries or pre-existing conditions not directly resulting from combat phases, and athletes who withdrew from the study. The subjects competed in 128 international tournaments of the European Judo Union (EJU) in 2005–2020, including European Judo Championships, and were aged between 19 and 35 years [16]. The study sample included all judo weight categories. The EJU injury registration form previously approved by the EJU Medical Commission and an informed consent form were signed by the athletes. They were also briefed about the procedure process related to the EJU injury registration form.

European Judo Union (EJU) medical data were used as the research material (195 injured judokas, including 93 women and 102 men). The inclusion criteria for the injury to be recorded for the study were as follows: (a) the injury was recorded in the records of the EJU and (b) the injury was diagnosed by a sports physician. The study sample was compiled using a systematic selection of athletes from the European Judo Union (EJU) injury database to ensure comprehensive representation. Participants were included based on pre-defined inclusion and exclusion criteria outlined in collaboration with the EJU Medical Commission. The participants were informed about the study aims, procedures, and data confidentiality during pre-competition briefings. Written informed consent was obtained in accordance with the guidelines of the Declaration of Helsinki. The study protocol for the retrospective analysis of data was approved by the Bioethics Committee at the Regional Medical Board (No. 287/KBL/OIL/2020).

The EJU medical questionnaire was utilized to gather information from each injured athlete according to the aforementioned inclusion criteria. Injuries were divided into the following 2 main categories: (a) common, not affecting the course of the fight (i.e., nose bleeds, bruises, and abrasions) and (b) serious (requiring medical intervention and resulting in fight discontinuation). Data were analyzed with the distinction of attacking and defending athletes in regard to sex and the place the injury occurred. The EJU medical questionnaire, adapted from standardized sports injury recording tools, was validated by the EJU Medical Commission for use in international judo competitions. It comprised sections detailing the injury location, type, severity, and circumstances of occurrence.

Injuries were recorded by on-site medical staff immediately after each combat phase and subsequently verified by licensed sports physicians within 24 h. These physicians, experienced in diagnosing judo-specific injuries, underwent specialized training provided by the EJU to ensure consistency in injury assessment and reporting. Verification involved a standardized injury diagnosis protocol, which included a physical examination and, when necessary, imaging studies to confirm the nature and extent of the injuries.

To maintain data quality, the collection process was supervised by senior medical staff and reviewed within 24 h by a second physician. Periodic audits by the EJU Medical Commission further ensured adherence to standardized procedures, and match video reviews were conducted to address any discrepancies. Reliability tests conducted during pilot events demonstrated a Cohen’s kappa coefficient of 0.85, indicating strong agreement among medical personnel.

Injuries were classified into the following three categories based on severity:

1. Minor—Injuries requiring first aid with no match withdrawal.
2. Moderate—Injuries necessitating medical intervention, potentially resulting in match withdrawal.
3. Severe—Injuries requiring advanced care and leading to tournament withdrawal.

In cases where athletes sustained multiple injuries during a single match or throughout the tournament, each injury was treated as a separate data point in the analysis. This approach enabled a comprehensive understanding of injury patterns, locations, and timing. To address the potential clustering effect of multiple injuries per athlete, statistical adjustments, including repeated measures analyses, were applied to control for within-subject correlations. Additionally, the dataset was meticulously reviewed to ensure that no injuries were double-counted. Each injury was uniquely coded based on the athlete, match phase, and injury type.

### 2.2. Statistical Analysis

The statistical tests were selected based on the nature of the data and the study objectives. The Shapiro–Wilk test was used to verify the normality of the data distribution, and Levene’s test assessed homogeneity of variance. All variable variances were found to have normal distributions with slight left- or right-skewed deviations, which remained within acceptable limits. Additionally, Mauchley’s test of sphericity was conducted, and since the results were not statistically significant, the assumption of sphericity was met.

For comparisons between combat phases and gender-specific differences, repeated measures ANOVA was employed due to its ability to handle within-subject variability. Post hoc analyses were conducted when significant main effects were observed to identify specific group differences. To control for the increased risk of Type I errors arising from multiple comparisons, Bonferroni corrections were applied where appropriate. This conservative approach ensured the robustness of statistical inferences.

Missing data points, such as incomplete injury reports, were addressed using multiple imputation methods to minimize potential bias and maintain statistical power. Sensitivity analyses confirmed that the imputed values did not significantly alter the results, ensuring that the dataset remained robust and representative of the study population. In order to calculate numerical percentage characteristics, contingency tables were used. Effect sizes were calculated to provide additional context for the statistical findings, with the partial eta squared (ηp2\eta_p^2ηp2) reported for the ANOVA tests to quantify the proportion of variance explained by each factor. All analyses adopted a significance level of *p* < 0.05, *p* < 0.05, *p* < 0.05. Statistical analyses were performed using Statistica 13.3 (TIBCO Software Inc., Palo Alto, CA, USA).

## 3. Results

The results shown in Table 1 denote that males suffered more injuries during the first and second half of combat compared to females, however, during the last minute of combat, the number of injuries was greater among women. During the golden score, among both men and women, an equal number of injuries was recorded.

As presented in Table 2, the majority of injuries occurred during the combined period of the last half and the last minute of the combat. The differences in the number of injuries between the first half and the sum total of the last half and the last minute of the combat were statistically significant (*p* < 0.001) in males and females.

Table 3, Table 4, Table 5, Table 6, Table 7 and Table 8 present the differences in the number of injuries between particular combat phases, with special consideration of the location of injury in males and females (Table 3), in accordance with the location of injury in females (Table 4), in accordance with the location of injury in males (Table 5), in accordance with the location of injury in males and females and the sum total of the last half and the last minute of the combat (Table 6), with the location of injury in females and the sum total of the last half and the last minute of the combat (Table 7), and with the location of injury in males and the sum total of the last half and the last minute of the combat (Table 8).

## 4. Discussion

The main finding of this study is that, during particular periods of a judo combat, the occurrence of injuries is greater. The obtained results are not in line with our hypothesis. This study shows that the number of injuries during the second half of the judo match is not significantly greater compared to the first half. However, during the combined period of the second half and the last minute of the combat, significantly more injuries occur compared to both the first half of the combat and the golden score. Such results were observed both among males and females. However, additional differences were recorded in accordance with sex. Although males suffered more injuries during the first and second half of combat compared to females, during the last minute of combat, the occurrence of injuries was greater among females.

To the best of the authors’ knowledge, this study is the first to address the issue of injuries in relation to the time structure during singular judo combat, which limits the possibility of result comparison. In judo, the work time is greater than the restitution time, and it may be characterized as high-intensity, anaerobic lactate activity [15,23,24]. Moreover, as reported by Thomas et al. [24], values of 85–90% of HRmax are typically observed during matches, further, lactate increases proportionally to activity duration. In the present study, a greater rate of injury occurrence towards the end of the judo contest (particularly during the last minute of combat) was recorded. This result seems warranted considering the physiological demands of the sport, decreased force production, and increasing fatigue during the course of the combat [15,21,23,24]. Thus, it may be indicated that the physical preparation of an athlete, particularly in terms of anaerobic endurance, is of the utmost importance, not only considering the contest outcome [21], but also the safety of an athlete. Moreover, this issue seems to be of particular importance for female athletes, as more injuries were recorded during the last minute of combat in women compared to men. However, the accumulation of fatigue and a decrease in performance might not be the only mechanism responsible for such outcomes. It has been shown that during both eliminatory phases and finals compared to other phases of the tournament, a higher injury rate occurs [15]. Given the lower level of fatigue during eliminatory phases, also other factors (such as home advantage; [16]), which require further investigation and are beyond the scope of this study, might also impact injury development in relation to the time structure of judo combat.

Franchini et al. [21] reported that, during simulated judo combat, despite the growing physiological strain on an athlete (increased heart rate, blood lactate, and rate of perceived exertion), the frequency and the duration of high-intensity actions such as attacks, feints, or groundwork remain unchanged. However, the grip duration decreased as the only affected strength–endurance-related variable during simulated matches, suggesting its importance in regard to fatigue development. Such time motion characteristics of judo combat (a shorter grip duration with a concomitant equal duration of high-intensity actions) may also partially explain why defending (uke) compared to attacking (tori) athletes are more likely to suffer an injury during judo combat [14,25,26]. Further, as previous reports have shown that the lightest and heaviest judo athletes displayed different, unique characteristics (i.e., particularly in the attack, defense, groundwork, and pause phases; [15]), the influence of the weight category should also be considered. Given that judo competition injuries are associated with different combat situations, where rate and frequency vary between weight categories due to differences in fighting style [15,27], this aspect, which was beyond the scope of this study, should also be acknowledged.

Our analysis shows several differences in injury prevalence according to sex. In general, males suffered more injuries during the first and second half of combat compared to females, however, during the last minute of combat, the number of injuries was greater among women. The literature related to the influence of gender on injury occurrence remains contradictory [8,15,27,28]. Further, a systematic review by Pocceco et al. [27] pointed out a general data inconsistency regarding sex differences in relation to injury prevention in judo athletes, as some studies show a higher risk of injuries among men, while others among women [15,18,29]. Such discrepancies are likely due to the various ages of study participants, weight categories, or the type of tournament (national/international level). The reasons behind sex-based differences are most likely multifactorial, including physical and psychological responses, hormonal fluctuations, relative strength, and combat strategies [23,30,31,32]. For example, Miarka et al. [15] showed that female athletes executed a greater number of groundwork techniques compared to men [15,30,32]. Moreover, for males compared to females, the occurrence of injury is more often associated with contact with the opponent and with the mat [15,32]. Thus, the most common mechanism of injury may vary between the sexes, due to differences in fighting strategy/style, which, in turn, will result in the prevalence of a certain injury type. Interestingly, among females, there are more reported contusions and distortions and less fractures compared to men [8,15,32]. The presented manuscript also showed differences according to sex, thus being in line with previous findings. However, for the first time, such differences were shown in regard to the phase of the combat. Based on current data, trainers should be aware of the differences between sexes and pay particular attention to the last minute of the combat in regard to female athletes. This, in turn, may lead to the development of an improved tactical and physical preparation of athletes.

### Limitations

Beyond the strengths of this study, its limitations also need to be addressed. Although we included a large sample of both elite male and female judokas, we did not account for official regulation changes, as well as the age, weight, and training experience of the participants, which might have influenced the obtained results. For example, more training experience might result in an improved falling technique and, therefore, a lower possibility of injury occurrence. Thus, it is recommended that future studies also take into the account the age and the training experience of participants, as well as analyze data with regard to major regulation changes, such as in the Tokyo 2020 Olympics [9]. Most importantly, as shown by Miarka et al. [15], significantly more injuries occurred in the eliminatory and finals compared to the other phases of the competition, therefore, this factor should also be considered. Given that, during judo combat, athletes are classified according to their body mass [5], as well as the fact that the fighting style of judo may be different among weight categories [33], it is recommended that future studies take this issue into account.

## 5. Conclusions

This study revealed significant patterns in the timing and frequency of injuries during judo matches, with the combined second half and last minute of combat showing the highest injury rates. Sex-specific differences were also observed, as males sustained more injuries during the first and second halves, while females experienced a higher frequency of injuries during the last minute. These findings highlight the importance of combat-phase-specific strategies for injury prevention.

### Practical Implication

The study results provide valuable insights for coaches and strength and conditioning specialists. They suggest that the physical preparation of athletes, particularly in terms of their anaerobic endurance, may contribute to reducing injury risk, especially in the final phases of the match. Coaches are advised to pay particular attention to the last minute of the match in the context of injury prevention, especially among women, who showed a higher frequency of injuries during this phase. Implementing strategies focused on enhancing the anaerobic endurance and tactical preparation of athletes, considering the differences between sexes, may help to decrease the incidence of injuries and improve safety during competitions. Moreover, knowledge related to injury incidence and characteristics during judo tournaments may be obtained for organizers and medical personnel, which, in turn, may result in the better preparation and improved safety of an athlete in the case of emergencies [8].

*Specific Recommendations*

1. For Coaches and Trainers:
  ○ Incorporate anaerobic endurance training to mitigate fatigue-related injuries, particularly for female athletes during the final phases of combat.
  ○ Develop combat-phase-specific techniques and tactics to reduce injury risks during high-intensity periods, such as the last minute and golden score.
  ○ Focus on improving defensive skills (e.g., falls and grip techniques) to minimize the likelihood of injuries sustained while under attack.
2. For Medical and Tournament Staff:
  ○ Enhance on-site medical preparedness, particularly during the second half and last-minute phases, where injury rates peak.
  ○ Utilize real-time monitoring systems to identify early signs of fatigue-related risk factors during matches.
3. For Policy Makers:
  ○ Consider revising competition rules to address high-risk scenarios, such as extended golden score periods, which may exacerbate fatigue and injury rates.

*Future Research Directions*

1. Broader Population Analysis:
  ○ Expand studies to include different age groups, weight categories, and training levels to provide a more comprehensive understanding of injury patterns.
2. Mechanisms of Injury:
  ○ Investigate the biomechanical and physiological factors contributing to combat-phase-specific injuries to refine prevention strategies.
3. Impact of Regulatory Changes:
  ○ Examine how changes in judo competition rules (e.g., weight categories and combat duration) influence injury rates and patterns.
4. Longitudinal Studies:
  ○ Conduct long-term studies to evaluate the effectiveness of phase-specific injury prevention strategies in reducing injury incidence and severity.

## Figures and Tables

**Table 1:** Differences in the number of injuries between particular combat phases.

	01 First Half 1	02 Last Half 2	03 Last Minute 3	04 Golden Score 4	D (1–2)	D (1–3)	D (1–4)	D (2–3)	D (2–4)	D (3–4)
F&M	67 (26.4)	93 (36.6)	84 (33.1)	10 (3.9)	−26 (−10.2) *p* = 0.1723	−17 (−6.7) *p* = 0.3732	57 (22.4) *p* = 0.1183	9 (3.5) *p* = 0.6218	83 (32.7) ***p* = 0.0374**	74 (29.1) *p* = 0.0573
F	27 (23.1)	38 (32.5)	47 (40.2)	5 (4.3)	−11 (−9.4) *p* = 0.4084	−20 (−17.1) *p* = 0.135	22 (18.8) *p* = 0.3354	−9 (−7.7) *p* = 0.4647	33 (28.2) *p* = 0.1922	42 (35.9) *p* = 0.1134
M	40 (29.2)	55 (40.2)	37 (27)	5 (3.7)	−15 (−11) *p* = 0.2620	3 (2.2) *p* = 0.8460	35 (25.6) *p* = 0.2241	18 (13.1) *p* = 0.1947	50 (36.5) *p* = 0.1058	32 (23.4) *p* = 0.2525

D—difference; F—female; M—male; F&M—females and males combined. Numbers in the table represent numerical values describing a given category (e.g., number of injuries, summary values). Values in parentheses indicate the percentage share within a specific group or category unless otherwise specified in the table description, statistically significant values in bold.

**Table 2:** Differences in the number of injuries between particular combat phases (sum total of the last half and the last minute of the combat).

	01 First Half 1	Sum_LastHalf + LastMinute 2	04 Golden Score 3	D (1–2)	D (1–3)	D (2–3)
F&M	67 (26.4)	177 (69.7)	10 (3.9)	−110 (−43.3) ***p* < 0.001**	57 (22.4) *p* = 0.3927	167 (65.8) ***p* < 0.001**
F	27 (23.1)	85 (72.7)	5 (4.3)	−58 (−49.6) ***p* < 0.001**	22 (18.8) *p* = 0.3354	80 (68.4) ***p* = 0.0013**
M	40 (29.2)	92 (67.2)	5 (3.7)	−52 (−38) ***p* < 0.001**	35 (25.6) *p* = 0.2215	87 (63.5) ***p* = 0.004**

D—difference; F—female; M—male; F&M—females and males combined.

**Table 3:** Differences in the number of injuries between particular combat phases in accordance with the location of injury in males and females.

Injury	01 First Half 1	02 Last Half 2	03 Last Minute 3	04 Golden Score 4	D (1–2)	D (1–3)	D (1–4)	D (2–3)	D (2–4)	D (3–4)
Skull	2 (3)	6 (6.5)	6 (7.1)	1 (10)	−4 (−3.5)	−4 (−4.2)	1 (−7)	0 (−0.7)	5 (−3.6)	5 (−2.9)
Face	1 (1.5)	4 (4.3)	5 (6)	1 (10)	−3 (−2.8)	−4 (−4.5)	0 (−8.5)	−1 (−1.7)	3 (−5.7)	4 (−4.1)
05 Eye	0 (0)	3 (3.2)	1 (1.2)	0 (0)	−3 (−3.2)	−1 (−1.2)	0 (0)	2 (2)	3 (3.2)	1 (1.2)
06 Ear	1 (1.5)	0 (0)	0 (0)	0 (0)	1 (1.5)	1 (1.5)	1 (1.5)	0 (0)	0 (0)	0 (0)
07 Nose	4 (6)	0 (0)	1 (1.2)	0 (0)	4 (6)	3 (4.8)	4 (6)	−1 (−1.2)	0 (0)	1 (1.2)
08 Mouth	3 (4.5)	2 (2.2)	2 (2.4)	0 (0)	1 (2.3)	1 (2.1)	3 (4.5)	0 (−0.2)	2 (2.2)	2 (2.4)
09 Neck	10 (14.9)	5 (5.4)	7 (8.3)	1 (10)	5 (9.6)	3 (6.6)	9 (4.9)	−2 (−3)	4 (−4.6)	6 (−1.7)
10 Throat	4 (6)	3 (3.2)	2 (2.4)	0 (0)	1 (2.7)	2 (3.6)	4 (6)	1 (0.9)	3 (3.2)	2 (2.4)
11 Clavicle/AC	5 (7.5)	6 (6.5)	5 (6)	0 (0)	−1 (1)	0 (1.5)	5 (7.5)	1 (0.5)	6 (6.5)	5 (6)
12 Shoulder/Arm	5 (7.5)	9 (9.7)	6 (7.1)	0 (0)	−4 (−2.2)	−1 (0.3)	5 (7.5)	3 (2.5)	9 (9.7)	6 (7.1)
13 Elbow joint	8 (11.9)	14 (15.1)	14 (16.7)	0 (0)	−6 (−3.1)	−6 (−4.7)	8 (11.9)	0 (−1.6)	14 (15.1)	14 (16.7)
14 Forearm	0 (0)	1 (1.1)	1 (1.2)	0 (0)	−1 (−1.1)	−1 (−1.2)	0 (0)	0 (−0.1)	1 (1.1)	1 (1.2)
15 Wrist	2 (3)	2 (2.2)	0 (0)	0 (0)	0 (0.8)	2 (3)	2 (3)	2 (2.2)	2 (2.2)	0 (0)
16 Hand/Finger	3 (4.5)	7 (7.5)	3 (3.6)	0 (0)	−4 (−3.1)	0 (0.9)	3 (4.5)	4 (4)	7 (7.5)	3 (3.6)
17 Thorax	3 (4.5)	5 (5.4)	1 (1.2)	1 (10)	−2 (−0.9)	2 (3.3)	2 (−5.5)	4 (4.2)	4 (−4.6)	0 (−8.8)
18 Back	1 (1.5)	0 (0)	2 (2.4)	0 (0)	1 (1.5)	−1 (−0.9)	1 (1.5)	−2 (−2.4)	0 (0)	2 (2.4)
19 Abdomen	0 (0)	0 (0)	0 (0)	0 (0)	0 (0)	0 (0)	0 (0)	0 (0)	0 (0)	0 (0)
20 Pelvis	0 (0)	0 (0)	0 (0)	1 (10)	0 (0)	0 (0)	−1 (−10)	0 (0)	−1 (−10)	−1 (−10)
23 Femur	2 (3)	2 (2.2)	1 (1.2)	0 (0)	0 (0.8)	1 (1.8)	2 (3)	1 (1)	2 (2.2)	1 (1.2)
24 Knee	9 (13.4)	14 (15.1)	18 (21.4)	4 (40)	−5 (−1.6)	−9 (−8)	5 (−26.6)	−4 (−6.4)	10 (−25)	14 (−18.6)
25 Leg	0 (0)	2 (2.2)	1 (1.2)	0 (0)	−2 (−2.2)	−1 (−1.2)	0 (0)	1 (1)	2 (2.2)	1 (1.2)
26 Ankle	1 (1.5)	4 (4.3)	4 (4.8)	1 (10)	−3 (−2.8)	−3 (−3.3)	0 (−8.5)	0 (−0.5)	3 (−5.7)	3 (−5.2)
27 Foot	3 (4.5)	4 (4.3)	1 (1.2)	0 (0)	−1 (0.2)	2 (3.3)	3 (4.5)	3 (3.1)	4 (4.3)	1 (1.2)
28 Other	0 (0)	0 (0)	2 (2.4)	0 (0)	0 (0)	−2 (−2.4)	0 (0)	−2 (−2.4)	0 (0)	2 (2.4)
29 None	0 (0)	0 (0)	1 (1.2)	0 (0)	0 (0)	−1 (−1.2)	0 (0)	−1 (−1.2)	0 (0)	1 (1.2)
Sum total	67 (26.4)	93 (36.6)	84 (33.1)	10 (3.9)	−26 (−10.2)	−17 (−6.7)	57 (22.4)	9 (3.5)	83 (32.7)	74 (29.1)
*p*-value (1–2)	*p*-value (1–3)		*p*-value (1–4)		*p*-value (2–3)		*p*-value (2–4)		*p*-value (3–4)	
*p* = 0.1723	*p* = 0.3732		*p* = 0.1183		*p* = 0.6218		***p* = 0.0374**		*p* = 0.0573	

**Table 4:** Differences in the number of injuries between particular combat phases in accordance with the location of injury in females.

Injury	01 First Half	02 Last Half	03 Last Minute	04 Golden Score	D (1–2)	D (1–3)	D (1–4)	D (2–3)	D (2–4)	D (3–4)
03 Skull	0 (0)	2 (5.3)	3 (6.4)	0 (0)	−2 (−5.3)	−3 (−6.4)	0 (0)	−1 (−1.1)	2 (5.3)	3 (6.4)
04 Face	0 (0)	0 (0)	0 (0)	0 (0)	0 (0)	0 (0)	0 (0)	0 (0)	0 (0)	0 (0)
05 Eye	0 (0)	0 (0)	1 (2.1)	0 (0)	0 (0)	−1 (−2.1)	0 (0)	−1 (−2.1)	0 (0)	1 (2.1)
06 Ear	1 (3.7)	0 (0)	0 (0)	0 (0)	1 (3.7)	1 (3.7)	1 (3.7)	0 (0)	0 (0)	0 (0)
07 Nose	0 (0)	0 (0)	1 (2.1)	0 (0)	0 (0)	−1 (−2.1)	0 (0)	−1 (−2.1)	0 (0)	1 (2.1)
08 Mouth	3 (11.1)	1 (2.6)	1 (2.1)	0 (0)	2 (8.5)	2 (9)	3 (11.1)	0 (0.5)	1 (2.6)	1 (2.1)
09 Neck	5 (18.5)	1 (2.6)	4 (8.5)	0 (0)	4 (15.9)	1 (10)	5 (18.5)	−3 (−5.9)	1 (2.6)	4 (8.5)
10 Throath	3 (11.1)	3 (7.9)	2 (4.3)	0 (0)	0 (3.2)	1 (6.9)	3 (11.1)	1 (3.6)	3 (7.9)	2 (4.3)
11 Clavicle/AC	1 (3.7)	3 (7.9)	3 (6.4)	0 (0)	−2 (−4.2)	−2 (−2.7)	1 (3.7)	0 (1.5)	3 (7.9)	3 (6.4)
12 Shoulder/Arm	2 (7.4)	3 (7.9)	2 (4.3)	0 (0)	−1 (−0.5)	0 (3.2)	2 (7.4)	1 (3.6)	3 (7.9)	2 (4.3)
13 Elbow joint	5 (18.5)	8 (21.1)	9 (19.2)	0 (0)	−3 (−2.5)	−4 (−0.6)	5 (18.5)	−1 (1.9)	8 (21.1)	9 (19.2)
14 Forearm	0 (0)	0 (0)	1 (2.1)	0 (0)	0 (0)	−1 (−2.1)	0 (0)	−1 (−2.1)	0 (0)	1 (2.1)
15 Wrist	0 (0)	1 (2.6)	0 (0)	0 (0)	−1 (−2.6)	0 (0)	0 (0)	1 (2.6)	1 (2.6)	0 (0)
16 Hand/Finger	2 (7.4)	3 (7.9)	1 (2.1)	0 (0)	−1 (−0.5)	1 (5.3)	2 (7.4)	2 (5.8)	3 (7.9)	1 (2.1)
17 Thorax	1 (3.7)	3 (7.9)	1 (2.1)	0 (0)	−2 (−4.2)	0 (1.6)	1 (3.7)	2 (5.8)	3 (7.9)	1 (2.1)
18 Back	0 (0)	0 (0)	2 (4.3)	0 (0)	0 (0)	−2 (−4.3)	0 (0)	−2 (−4.3)	0 (0)	2 (4.3)
19 Abdomen	0 (0)	0 (0)	0 (0)	0 (0)	0 (0)	0 (0)	0 (0)	0 (0)	0 (0)	0 (0)
20 Pelvis	0 (0)	0 (0)	0 (0)	1 (20)	0 (0)	0 (0)	−1 (−20)	0 (0)	−1 (−20)	−1 (−20)
23 Femur	0 (0)	1 (2.6)	0 (0)	0 (0)	−1 (−2.6)	0 (0)	0 (0)	1 (2.6)	1 (2.6)	0 (0)
24 Knee	3 (11.1)	6 (15.8)	12 (25.5)	3 (60)	−3 (−4.7)	−9 (−14.4)	0 (−48.9)	−6 (−9.7)	3 (−44.2)	9 (−34.5)
25 Leg	0 (0)	0 (0)	0 (0)	0 (0)	0 (0)	0 (0)	0 (0)	0 (0)	0 (0)	0 (0)
26 Ankle	1 (3.7)	2 (5.3)	3 (6.4)	1 (20)	−1 (−1.6)	−2 (−2.7)	0 (−16.3)	−1 (−1.1)	1 (−14.7)	2 (−13.6)
27 Foot	0 (0)	1 (2.6)	1 (2.1)	0 (0)	−1 (−2.6)	−1 (−2.1)	0 (0)	0 (0.5)	1 (2.6)	1 (2.1)
Sum total	27 (23.1)	38 (32.5)	47 (40.2)	5 (4.3)	−11 (−9.4)	−20 (−17.1)	22 (18.8)	−9 (−7.7)	33 (28.2)	42 (35.9)
*p*-value (1–2)	*p*-value (1–3)		*p*-value (1–4)		*p*-value (2–3)		*p*-value (2–4)		*p*-value (3–4)	
*p* = 0.4084	*p* = 0.1350		*p* = 0.3354		*p* = 0.4647		*p* = 0.1922		*p* = 0.1134	

D—difference; F—female; M—male; F&M—females and males combined.

**Table 5:** Differences in the number of injuries between particular combat phases in accordance with the location of injury in males.

Injury	01 First Half	02 Last Half	03 Last Minute	04 Golden Score	D (1–2)	D (1–3)	D (1–4)	D (2–3)	D (2–4)	D (3–4)
03 Skull	2 (5)	4 (7.3)	3 (8.1)	1 (20)	−2 (−2.3)	−1 (−3.1)	1 (−15)	1 (−0.8)	3 (−12.7)	2 (−11.9)
04 Face	1 (2.5)	4 (7.3)	5 (13.5)	1 (20)	−3 (−4.8)	−4 (−11)	0 (−17.5)	−1 (−6.2)	3 (−12.7)	4 (−6.5)
05 Eye	0 (0)	3 (5.5)	0 (0)	0 (0)	−3 (−5.5)	0 (0)	0 (0)	3 (5.5)	3 (5.5)	0 (0)
06 Ear	0 (0)	0 (0)	0 (0)	0 (0)	0 (0)	0 (0)	0 (0)	0 (0)	0 (0)	0 (0)
07 Nose	4 (10)	0 (0)	0 (0)	0 (0)	4 (10)	4 (10)	4 (10)	0 (0)	0 (0)	0 (0)
08 Mouth	0 (0)	1 (1.8)	1 (2.7)	0 (0)	−1 (−1.8)	−1 (−2.7)	0 (0)	0 (−0.9)	1 (1.8)	1 (2.7)
09 Neck	5 (12.5)	4 (7.3)	3 (8.1)	1 (20)	1 (5.2)	2 (4.4)	4 (−7.5)	1 (−0.8)	3 (−12.7)	2 (−11.9)
10 Throath	1 (2.5)	0 (0)	0 (0)	0 (0)	1 (2.5)	1 (2.5)	1 (2.5)	0 (0)	0 (0)	0 (0)
11 Clavicle/AC	4 (10)	3 (5.5)	2 (5.4)	0 (0)	1 (4.6)	2 (4.6)	4 (10)	1 (0)	3 (5.5)	2 (5.4)
12 Shoulder/Arm	3 (7.5)	6 (10.9)	4 (10.8)	0 (0)	−3 (−3.4)	−1 (−3.3)	3 (7.5)	2 (0.1)	6 (10.9)	4 (10.8)
13 Elbow joint	3 (7.5)	6 (10.9)	5 (13.5)	0 (0)	−3 (−3.4)	−2 (−6)	3 (7.5)	1 (−2.6)	6 (10.9)	5 (13.5)
14 Forearm	0 (0)	1 (1.8)	0 (0)	0 (0)	−1 (−1.8)	0 (0)	0 (0)	1 (1.8)	1 (1.8)	0 (0)
15 Wrist	2 (5)	1 (1.8)	0 (0)	0 (0)	1 (3.2)	2 (5)	2 (5)	1 (1.8)	1 (1.8)	0 (0)
16 Hand/Finger	1 (2.5)	4 (7.3)	2 (5.4)	0 (0)	−3 (−4.8)	−1 (−2.9)	1 (2.5)	2 (1.9)	4 (7.3)	2 (5.4)
17 Thorax	2 (5)	2 (3.6)	0 (0)	1 (20)	0 (1.4)	2 (5)	1 (−15)	2 (3.6)	1 (−16.4)	−1 (−20)
18 Back	1 (2.5)	0 (0)	0 (0)	0 (0)	1 (2.5)	1 (2.5)	1 (2.5)	0 (0)	0 (0)	0 (0)
19 Abdomen	0 (0)	0 (0)	0 (0)	0 (0)	0 (0)	0 (0)	0 (0)	0 (0)	0 (0)	0 (0)
20 Pelvis	0 (0)	0 (0)	0 (0)	0 (0)	0 (0)	0 (0)	0 (0)	0 (0)	0 (0)	0 (0)
23 Femur	2 (5)	1 (1.8)	1 (2.7)	0 (0)	1 (3.2)	1 (2.3)	2 (5)	0 (−0.9)	1 (1.8)	1 (2.7)
24 Knee	6 (15)	8 (14.6)	6 (16.2)	1 (20)	−2 (0.5)	0 (−1.2)	5 (−5)	2 (−1.7)	7 (−5.5)	5 (−3.8)
25 Leg	0 (0)	2 (3.6)	1 (2.7)	0 (0)	−2 (−3.6)	−1 (−2.7)	0 (0)	1 (0.9)	2 (3.6)	1 (2.7)
26 Ankle	0 (0)	2 (3.6)	1 (2.7)	0 (0)	−2 (−3.6)	−1 (−2.7)	0 (0)	1 (0.9)	2 (3.6)	1 (2.7)
27 Foot	3 (7.5)	3 (5.5)	0 (0)	0 (0)	0 (2.1)	3 (7.5)	3 (7.5)	3 (5.5)	3 (5.5)	0 (0)
28 Other	0 (0)	0 (0)	2 (5.4)	0 (0)	0 (0)	−2 (−5.4)	0 (0)	−2 (−5.4)	0 (0)	2 (5.4)
29 None	0 (0)	0 (0)	1 (2.7)	0 (0)	0 (0)	−1 (−2.7)	0 (0)	−1 (−2.7)	0 (0)	1 (2.7)
Sum total	40 (29.2)	55 (40.2)	37 (27)	5 (3.7)	−15 (−11)	3 (2.2)	35 (25.6)	18 (13.1)	50 (36.5)	32 (23.4)
*p*-value (1–2)	*p*-value (1–3)		*p*-value (1–4)		*p*-value (2–3)		*p*-value (2–4)		*p*-value (3–4)	
*p* = 0.2620	*p* = 0.8460		*p* = 0.2241		*p* = 0.1947		*p* = 0.1058		*p* = 0.2525	

**Table 6:** Differences in the number of injuries between particular combat phases in accordance with the location of injury in males and females (sum total of the last half and the last minute of the combat).

Injury	01 First Half 1	Sum_LastHalf + LastMinute 2	04 Golden Score 3	D (1–2)	D (1–3)	D (2–3)
03 Skull	2 (3)	12 (6.8)	1 (10)	−10 (−3.8)	1 (−7)	11 (−3.2)
04 Face	1 (1.5)	9 (5.1)	1 (10)	−8 (−3.6)	0 (−8.5)	8 (−4.9)
05 Eye	0 (0)	4 (2.3)	0 (0)	−4 (−2.3)	0 (0)	4 (2.3)
06 Ear	1 (1.5)	0 (0)	0 (0)	1 (1.5)	1 (1.5)	0 (0)
07 Nose	4 (6)	1 (0.6)	0 (0)	3 (5.4)	4 (6)	1 (0.6)
08 Mouth	3 (4.5)	4 (2.3)	0 (0)	−1 (2.2)	3 (4.5)	4 (2.3)
09 Neck	10 (14.9)	12 (6.8)	1 (10)	−2 (8.2)	9 (4.9)	11 (−3.2)
10 Throath	4 (6)	5 (2.8)	0 (0)	−1 (3.2)	4 (6)	5 (2.8)
11 Clavicle/AC	5 (7.5)	11 (6.2)	0 (0)	−6 (1.3)	5 (7.5)	11 (6.2)
12 Shoulder/Arm	5 (7.5)	15 (8.5)	0 (0)	−10 (−1)	5 (7.5)	15 (8.5)
13 Elbow joint	8 (11.9)	28 (15.8)	0 (0)	−20 (−3.9)	8 (11.9)	28 (15.8)
14 Forearm	0 (0)	2 (1.1)	0 (0)	−2 (−1.1)	0 (0)	2 (1.1)
15 Wrist	2 (3)	2 (1.1)	0 (0)	0 (1.9)	2 (3)	2 (1.1)
16 Hand/Finger	3 (4.5)	10 (5.7)	0 (0)	−7 (−1.2)	3 (4.5)	10 (5.7)
17 Thorax	3 (4.5)	6 (3.4)	1 (10)	−3 (1.1)	2 (−5.5)	5 (−6.6)
18 Back	1 (1.5)	2 (1.1)	0 (0)	−1 (0.4)	1 (1.5)	2 (1.1)
19 Abdomen	0 (0)	0 (0)	0 (0)	0 (0)	0 (0)	0 (0)
20 Pelvis	0 (0)	0 (0)	1 (10)	0 (0)	−1 (−10)	−1 (−10)
23 Femur	2 (3)	3 (1.7)	0 (0)	−1 (1.3)	2 (3)	3 (1.7)
24 Knee	9 (13.4)	32 (18.1)	4 (40)	−23 (−4.7)	5 (−26.6)	28 (−21.9)
25 Leg	0 (0)	3 (1.7)	0 (0)	−3 (−1.7)	0 (0)	3 (1.7)
26 Ankle	1 (1.5)	8 (4.5)	1 (10)	−7 (−3)	0 (−8.5)	7 (−5.5)
27 Foot	3 (4.5)	5 (2.8)	0 (0)	−2 (1.7)	3 (4.5)	5 (2.8)
28 Other	0 (0)	2 (1.1)	0 (0)	−2 (−1.1)	0 (0)	2 (1.1)
29 None	0 (0)	1 (0.6)	0 (0)	−1 (−0.6)	0 (0)	1 (0.6)
Sum total	67 (26.4)	177 (69.7)	10 (3.9)	−110 (−43.3)	57 (22.4)	167 (65.8)
*p*-value (1–2)		*p*-value (1–3)		*p*-value (2–3)		
***p* < 0.001**		*p* = 0.3927		***p* < 0.001**		

**Table 7:** Differences in the number of injuries between particular combat phases in accordance with the location of injury in females (sum total of the last half and the last minute of the combat).

Injury	01 First Half	Sum_LastHalf+ Last Minute	04 Golden Score	D (1–2)	D (1–3)	D (2–3)
03 Skull	0 (0)	5 (5.9)	0 (0)	−5 (−5.9)	0 (0)	5 (5.9)
04 Face	0 (0)	0 (0)	0 (0)	0 (0)	0 (0)	0 (0)
05 Eye	0 (0)	1 (1.2)	0 (0)	−1 (−1.2)	0 (0)	1 (1.2)
06 Ear	1 (3.7)	0 (0)	0 (0)	1 (3.7)	1 (3.7)	0 (0)
07 Nose	0 (0)	1 (1.2)	0 (0)	−1 (−1.2)	0 (0)	1 (1.2)
08 Mouth	3 (11.1)	2 (2.4)	0 (0)	1 (8.8)	3 (11.1)	2 (2.4)
09 Neck	5 (18.5)	5 (5.9)	0 (0)	0 (12.6)	5 (18.5)	5 (5.9)
10 Throath	3 (11.1)	5 (5.9)	0 (0)	−2 (5.2)	3 (11.1)	5 (5.9)
11 Clavicle/AC	1 (3.7)	6 (7.1)	0 (0)	−5 (−3.4)	1 (3.7)	6 (7.1)
12 Shoulder/Arm	2 (7.4)	5 (5.9)	0 (0)	−3 (1.5)	2 (7.4)	5 (5.9)
13 Elbow joint	5 (18.5)	17 (20)	0 (0)	−12 (−1.5)	5 (18.5)	17 (20)
14 Forearm	0 (0)	1 (1.2)	0 (0)	−1 (−1.2)	0 (0)	1 (1.2)
15 Wrist	0 (0)	1 (1.2)	0 (0)	−1 (−1.2)	0 (0)	1 (1.2)
16 Hand/Finger	2 (7.4)	4 (4.7)	0 (0)	−2 (2.7)	2 (7.4)	4 (4.7)
17 Thorax	1 (3.7)	4 (4.7)	0 (0)	−3 (−1)	1 (3.7)	4 (4.7)
18 Back	0 (0)	2 (2.4)	0 (0)	−2 (−2.4)	0 (0)	2 (2.4)
19 Abdomen	0 (0)	0 (0)	0 (0)	0 (0)	0 (0)	0 (0)
20 Pelvis	0 (0)	0 (0)	1 (20)	0 (0)	−1 (−20)	−1 (−20)
23 Femur	0 (0)	1 (1.2)	0 (0)	−1 (−1.2)	0 (0)	1 (1.2)
24 Knee	3 (11.1)	18 (21.2)	3 (60)	−15 (−10.1)	0 (−48.9)	15 (−38.8)
25 Leg	0 (0)	0 (0)	0 (0)	0 (0)	0 (0)	0 (0)
26 Ankle	1 (3.7)	5 (5.9)	1 (20)	−4 (−2.2)	0 (−16.3)	4 (−14.1)
27 Foot	0 (0)	2 (2.4)	0 (0)	−2 (−2.4)	0 (0)	2 (2.4)
Sum total	27 (23.1)	85 (72.7)	5 (4.3)	−58 (−49.6)	22 (18.8)	80 (68.4)
*p*-value (1–2)		*p*-value (1–3)		*p*-value (2–3)		
***p* < 0.001**		*p* = 0.3354		***p* = 0.0013**		

**Table 8:** Differences in the number of injuries between particular combat phases in accordance with the location of injury in males (sum total of the last half and the last minute of the combat).

Injury	01 First Half	Sum_LastHalf + LastMinute	04 Golden Score	D (1–2)	D (1–3)	D (2–3)
03 Skull	2 (5)	7 (7.6)	1 (20)	−5 (−2.6)	1 (−15)	6 (−12.4)
04 Face	1 (2.5)	9 (9.8)	1 (20)	−8 (−7.3)	0 (−17.5)	8 (−10.2)
05 Eye	0 (0)	3 (3.3)	0 (0)	−3 (−3.3)	0 (0)	3 (3.3)
06 Ear	0 (0)	0 (0)	0 (0)	0 (0)	0 (0)	0 (0)
07 Nose	4 (10)	0 (0)	0 (0)	4 (10)	4 (10)	0 (0)
08 Mouth	0 (0)	2 (2.2)	0 (0)	−2 (−2.2)	0 (0)	2 (2.2)
09 Neck	5 (12.5)	7 (7.6)	1 (20)	−2 (4.9)	4 (−7.5)	6 (−12.4)
10 Throath	1 (2.5)	0 (0)	0 (0)	1 (2.5)	1 (2.5)	0 (0)
11 Clavicle/AC	4 (10)	5 (5.4)	0 (0)	−1 (4.6)	4 (10)	5 (5.4)
12 Shoulder/Arm	3 (7.5)	10 (10.9)	0 (0)	−7 (−3.4)	3 (7.5)	10 (10.9)
13 Elbow joint	3 (7.5)	11 (12)	0 (0)	−8 (−4.5)	3 (7.5)	11 (12)
14 Forearm	0 (0)	1 (1.1)	0 (0)	−1 (−1.1)	0 (0)	1 (1.1)
15 Wrist	2 (5)	1 (1.1)	0 (0)	1 (3.9)	2 (5)	1 (1.1)
16 Hand/Finger	1 (2.5)	6 (6.5)	0 (0)	−5 (−4)	1 (2.5)	6 (6.5)
17 Thorax	2 (5)	2 (2.2)	1 (20)	0 (2.8)	1 (−15)	1 (−17.8)
18 Back	1 (2.5)	0 (0)	0 (0)	1 (2.5)	1 (2.5)	0 (0)
19 Abdomen	0 (0)	0 (0)	0 (0)	0 (0)	0 (0)	0 (0)
20 Pelvis	0 (0)	0 (0)	0 (0)	0 (0)	0 (0)	0 (0)
23 Femur	2 (5)	2 (2.2)	0 (0)	0 (2.8)	2 (5)	2 (2.2)
24 Knee	6 (15)	14 (15.2)	1 (20)	−8 (−0.2)	5 (−5)	13 (−4.8)
25 Leg	0 (0)	3 (3.3)	0 (0)	−3 (−3.3)	0 (0)	3 (3.3)
26 Ankle	0 (0)	3 (3.3)	0 (0)	−3 (−3.3)	0 (0)	3 (3.3)
27 Foot	3 (7.5)	3 (3.3)	0 (0)	0 (4.2)	3 (7.5)	3 (3.3)
28 Other	0 (0)	2 (2.2)	0 (0)	−2 (−2.2)	0 (0)	2 (2.2)
29 None	0 (0)	1 (1.1)	0 (0)	−1 (−1.1)	0 (0)	1 (1.1)
Sum total	40 (29.2)	92 (67.2)	5 (3.7)	−52 (−38)	35 (25.6)	87 (63.5)
*p*-value (1–2)		*p*-value (1–3)		*p*-value (2–3)		
***p* < 0.001**		*p* = 0.2215		***p* = 0.004**		

## Data Availability

All data were included in the manuscript.
